# Supplementary material for: A microRNA Prognostic Signature in Patients with Diffuse Intrinsic Pontine Gliomas through Non-Invasive Liquid Biopsy
Source: Cancers (Basel). 2022 Sep 2;14(17):4307. doi: 10.3390/cancers14174307 (PMC9454461; doi:10.3390/cancers14174307)
Supplement: Supplementary file 1 [file cancers-14-04307-s001.zip › edited-Supplementary Methods_rev.pdf]

Supplementary Methods

Study material

Fifty-two patients with newly-diagnosed DIPG were retrospectively enrolled, whose serum samples were collected at the baseline, before any treatment. Five patients were later excluded: in four cases, this was due to high levels of hemolysis in their serum samples, while one case was excluded after MRI revision and H3K27M-mutation analysis. DIPG diagnosis was based on a centralized MRI images review and only in case of uncertainties in radiological diagnosis, a biopsy was required. In our case series, five patients had undergone stereotactic or open surgical biopsy before being enrolled for any treatment because of atypical radiological findings. Four of these cases had been confirmed as H3K27M-mutant DMG, using immunohistochemistry or a PCR-RFLP strategy,15 while the sole case not revealing the H3K27M mutation was excluded.

After DIPG initial diagnosis by a central radiological review at the time of enrolment for the trial, the diagnosis was confirmed by a second neuroradiologist blinded to patients' clinical data.

Eventually, the present study enrolled 47 cases: 23 as "training set"; and 24 cases, which served as the "validation set".

Eligibility Criteria

Inclusion Criteria:

- Patients from 2 to 21 years old will be eligible
- No previous treatment consented apart from steroids
- Strict eligibility criteria will radiologically-verified DIPG (an intrinsic, pontine-based infiltrative lesion hypointense on T1- and hyperintense on T2-weighted sequences, involving at least 2/3 of the pons)
- Symptoms lasting less than 6 months
- Life expectancy ≥4 weeks

- Karnowski/Lansky performance status ≥ 40 %
- No organ dysfunction; no pregnancy or breast-feeding
- Patients undergo baseline cranial MRI with gadolinium, to be repeated if treatment begins more than 2 weeks; spinal MRI due to the occurrence of metastatic cases at diagnosis will also be mandatory
- Written and signed informed consent from parents or legal guardians will be obtained before starting the treatment.

Exclusion Criteria:

- Patients below 2 years or over 21
- Pre-treatment with radio or chemotherapy
- Neurofibromatosis 1
- Non-typical imaging
- Symptoms duration over 6 months, Lansky/Karnowski scores below 40%
- Metastatic disease as shown by MRI
- Organ dysfunction, pregnancy or breast-feeding
- Absence of parents, patient or tutor consent

Treatment schedule

The treatment schedule for the training and validation data sets is detailed below.

Training set. Nimotuzumab and vinorelbine infusions were scheduled as follows[19]: (i) 150 mg /m²/d of nimotuzumab weekly, and 20 mg/m²/d of vinorelbine weekly, in weeks 1 to 12, in short 30-min iv. infusions (induction phase); (ii) first reassessment at week 12 (day 84–92) and, in cases without disease progression, nimotuzumab 150 mg/m²/d and vinorelbine 25 mg/m²/d administered in weeks 14, 16, 18, 20, 22, and 24 (consolidation phase I); (iii) second reassessment at week 24, in cases without disease progression: nimotuzumab 150 mg/m²/d and vinorelbine 25 mg/m²/d given fortnightly. All cases were then reassessed at week 36, and every 12 weeks thereafter, and given the

same systemic treatment until progression, or up to week 104. Radiotherapy was scheduled to begin within 3 weeks of starting the therapy with nimotuzumab and vinorelbine. A total dose of 54 Gy, conventionally fractionated (1.8 Gy/fraction, 5 days a week), was delivered by a 6 MV linear accelerator.

Validation set. A prospective, non-blinded, randomized two-cohort study was approved at our institution and is currently underway (INT-DIPG2015). Patients enrolled in that study were randomized, to form two arms: (a) one followed the standard protocol, as described elsewhere[19]; (b) the other, experimental arm was treated with three courses of RT: a first 36 Gy applied to the tumor bed in 20 daily fractions, alongside with the above-described systemic nimotuzumab and vinorelbine treatment; next, two re-irradiation courses of 19.8 Gy (1.8 Gy/fr) starting from weeks 25 and 45, while continuing to receive the same systemic treatment. To be consistent with the training set, the validation set only included samples from patients enrolled in the first arm (i.e., those treated with the standard protocol).

Sample size calculation

Considering the findings obtained in the training set, the sample size for prospective validation purposes was calculated based on the number of events required to reach relative hazard between high and low risk patient groups of 4. Imposing α (two-tailed) = 0.05, power ($1-\beta$) = 0.9, a proportion of subjects in each class (i.e. high or low risk) = 0.5, and RH (relative hazard) = 4, the total events needed for validation purposes is 22. The enrolled validation cohort fulfills the sample size requirement having n=24 and adequate follow-up with progression experienced by all patient. Calculation are based on UCSF's sample-size web-tool (Kohn and Senyak, 2021).

Sample processing and RNA extraction

Blood samples were collected at the baseline, kept at 4°C for 30 min, then processed within 1 h after collection by centrifugation at 1600 × *g* at 4°C for 10 min. Serum was aliquoted in vials

and stored at –80°C. Because hemolysis levels reportedly may affect microRNA profiles (Kirschner, 2013), we checked whether all the serum samples met the criteria for subsequent analyses while excluding any analytical biases. A hemolysis score was derived according to Appierto et al (Appierto, 2014), using a spectrophotometrically-based system. Specifically, the spectrophotometric quantification of hemoglobin release was performed using the NanoDrop One (ThermoFisher) spectrophotometer, by measuring the absorbance spectra at 414 nm and 385 nm. The procedure required 2 µl of each serum sample on the micro-volume instrument's pedestal, for which a saline solution (PBS) was used as a blank. Total RNA from each sample (n = 47) was extracted from 200 µl of serum subsample using the miRCURY RNA Isolation Biofluid kit according to the manufacturer's protocols (Exiqon, Denmark). The MS2 carrier was added to the samples; after lysis of the biofluid components, protein precipitation, and DNA digestion by a DNAse, the RNA was purified in spin columns. The Usp6 spike-in was also added to each sample, to check the quality of the extraction process. Finally, the RNA-extracted samples were stored at –80°C.

Microarray profiling

The miRNA microarray experiments—performed on the Integrated Biology Platform at the Department of Applied Research and Technology Development (Fondazione IRCCS Istituto Tumori)—used Human miRNA SurePrint Microarrays (Agilent Technology) that contained 2006 miRNAs annotated in miRBase 19.0. For each plasma sample, total RNA was labeled, hybridized onto the miRNA array, and further processed following the manufacturer's protocols. The arrays were scanned by a G2565CA scanner (Agilent Technology) and the ensuing scanned images processed using Feature Extraction software v10.5 (Agilent Technology), to extract the raw data. Then the Bioconductor library AgiMicroRna (López-Romero P., 2011) was used to process this raw data of the 47 samples. Multiple probes were spotted onto the array for each miRNA, so the raw data underwent the following processing steps: i) removing those probes with a low detection rate

(i.e., not detected in at least 25% of the samples), ii) background adjustment; iii) quantile normalization using the Robust Multiarray Averaging (RMA) method (Irizarry, 2003), iv) subsequent log2-transformations. ComBat was applied, which adjusts the RNA extraction batches (Johnson, 2007), to reduce the likelihood of systemic, not biological but rather technical or experimental issues causing batch effects.

Microarray data compliant with the Minimum Information About a Microarray Experiment (MIAME) guidelines were deposited in the GEO (Gene Expression Omnibus) repository (http://www.ncbi.nlm.nih.gov/geo/), under accession number GSE160249.

The survival model's development and assessment of its performance

We first developed a survival signature using the training data set. A signature related to PFS was identified through a standardized, semi-supervised principal component method (Bair and Tibshirani, 2004). The features (i.e. miRNAs) entered into the signature were examined by applying a univariate Cox's proportional hazards regression on PFS as predicted by the miRNA log_2-transformed expression level. The aim was to obtain a model based on circulating miRNA expression profiles in our training set. To ensure it could produce a robust prediction for a new sample without overfitting by the algorithm, a leave-one-out cross-validation (LOO-CV) method was used to construct this model, which is recommended for the internal validation of small-sized sets (Abu Zohair, 2019).

After reiterating the whole procedure—leaving out a sample each time until all cases had been omitted once—the LOO-CV provided an estimate of the coefficient threshold for segregate cases by progression risk based on the median score value. Then the dimensionality of the miRNAs entering the model was reduced by a principal component analysis (PCA) to capture most of their variability. The first principal component was used to predict survival outcome, and to assign a prediction index to each patient. For this, a permutation test based on 100 fold resampling was computed to estimate the potential degree of overfitting; briefly, this entailed a multistep, random

shuffling of the survival data, with the cross-validation process repeated to measure the statistical significance of the association between circulating miRNA expression levels and survival data. The log-rank statistic was calculated for these permutations.

Since the aim was to determine the ability of circulating miRNAs' expression to predict PFS, for model validation purposes we designated the linear combination of our 13 miRNAs as a biomarker complex. Its index can be calculated this way:

$\sum^{i} w_i x_i - 6.810089$

where, w_i and x_i are respectively the weight and log-transformed gene expression level for the *i*-th miRNA, whose summation is scaled by a value (–6.810089) computed from the cross-validation process. A given sample is thus predicted to carry a *high risk* of progression when the score is > 0.007481, and conversely a *low risk* when it is ≤ 0.007481. The 13 miRNAs and their weights are listed in Supplementary Table 1.

The above analyses were carried out with the 'superpc' package in R (http://www-stat.stanford.edu/~tibs/superpc), integrated in BRB-ArrayTools v4.6 as an Excel plug-in, developed by Dr. Richard Simon and the BRB-ArrayTools Development Team (Simon, 2007).

Statistical analysis

Descriptive statistics of the ct-miRNA signature index scores obtained with the ct-miRNA model were examined for skewness (a measure of the asymmetry of the data distribution) and kurtosis (a measure of the tail-heaviness of the distribution), respectively computed with the D'Agostino and Anscombe-Glynn tests (Wessa, 2020). To test the normality of ct-miRNA signature, the Shapiro-Wilk's test through the *shapiro.test* R function was performed comparing the sample distribution to a normal one in order to ascertain whether data show or not a serious deviation from normality. From the output of *shapiro.test* function, the p-value > 0.05 implies that the data distribution is not significantly different from normal distribution, assuming normality. We also checked the distribution of residuals for detecting violation of standard regression assumptions by

ols_plot_resid_qq function in 'olsrr' package. Since we were interested in the ct-miRNA model's ability to stratify patients based on their survival data, we considered the bivariate kernel density estimation of the joint distribution function fitting the ct-miRNA signature, and the time-to-event variable, retaining the information from censored observations by using the *funcen* function available in the 'smoothROCtime' package. We applied the wireframe function for visualization purposes (Martínez-Camblor, 2018). Skewness and kurtosis are first-order statistical properties of the data distribution underpinning our proposed ct-miRNA model, while kernel density estimation is useful for depicting the distribution's heterogeneity and identifying the presence of multimodal shapes. The model's ability to predict survival risk was examined using Kaplan–Meier curves, whose statistical significance was assessed with the log-rank test, both implemented by the 'survival' package in R (Therneau, 2020). Stratification by risk for censored data was evaluated by applying a weighted log-rank test, the Fleming–Harrington test, using the score vector distributions. These tests were conducted under the *FHtesticp* function in the 'FHtest' package, in R, which uses a permutation approach based on asymptotic approximations of the CLT [central limit theorem] (Oller, 2017). Since the proportional hazard assumption is supported by a non-significant relationship between residuals and time, this was checked using a statistical test based on the scaled Schoenfeld residuals. The function *cox.zph* in the 'survival' package allows testing the proportional hazards assumption for each covariate included in a Cox regression model fit. The function *cox.zph* correlates the corresponding set of scaled Schoenfeld residuals with time, to test for independence between residuals and time.

Univariate Cox's proportional hazards regression was used to analyze the relationship between the ct-miRNA model [high vs low risk] and PFS or OS. We ran a multivariate Cox's regression to see whether our model provided more accurate predictions than other covariates (age and hydrocephalus). These results are presented as hazard ratios (HR) with their 95% confidence intervals and corresponding P-values for the two-sided Wald test, all obtained using the 'survival' package in R. Model checking and performance estimates were assessed in terms of: i) prediction

error, based on Brier scores; ii) calibration; iii) discrimination; and iv) decision curve analysis. We considered the landmark timepoints of 8.5 months for the primary endpoint (i.e. PFS) and 13.7 months for the secondary endpoint (i.e. OS) corresponding to the median progression and survival times, respectively, in the validation cohort. The established landmark timepoints were consistent with the results of the pilot phase 2 study that combined nimotuzumab with concomitant radiation and vinorelbine (Massimino, 2014) reporting 8.5 and 15 months as median PFS and OS, respectively.

Prediction error: The Brier score (Brier, 1950) gives an estimate of the mean squared error of a prediction. It is computed from a weighted average of time-dependent residuals, where the residual is defined as the difference between the time-dependent survival status (high vs. low risk) and the probability predicted from a Cox's regression model (Gerds and Schumacher, 2006, Porzelius 2009). This score's function is averaged over the whole follow-up period, yielding an overall Brier score. If the predicted survival status perfectly matched the actual (observed) status at each point during the follow-up period, then the Brier score would be zero. Further facilitating the Brier score's interpretation is that its benchmark value of 0.25 corresponds to a 50% risk prediction probability. The prediction error of the Brier score (Gerds, 2007) was computed here as a function of survival time, using the 'pec' package in R and the LOO-CV (Mogensen, 2012). The null reference scenario for the Brier score corresponded to a model without any predictor terms and covariate data. For visualization purposes, the prediction error curves of the miRNA model and that reference scenario were plotted over the follow-up period. To summarize the prediction error, an Integrated Brier Score (IBS) was computed; it provides the overall calculation of the model's performance at all available times, $t1 \leq t \leq t\text{max}$.

Calibration: A graphical method was also used to gauge the extent to which the predicted risks matched the observed events (relative frequency). This calibration plot can be drawn as jagged line by fitting a linear regression to observed proportions against predicted probabilities, yielding an intercept and slope: for a perfect fit, the plot should have a diagonal (45°) line, through the origin,

whose slope is 1. The *calPlot* function available in the 'pec package' in R (Mogensen, 2012) was used to draw the calibration plot at the landmark time of 8.5 months, the median follow-up for PFS, and that of 13.7 months, when the model was tested against OS.

Discrimination: Discrimination was examined using the time-dependent receiver operating characteristic (ROC) curves with censored data (Heagerty, 2000). This method provides a combined function of the sensitivity (true positive rate) and specificity (true negative rate) over time, with the area under the curve (AUC) conveying overall accuracy in predicting the outcome of interest. A Kaplan–Meier estimator of the censoring distribution was used, with the 'timeROC' package in R (Blanche, 2013), whose *timeROC* function derives time-dependent ROC curves for censored data, and this to test the AUC of our miRNA model over the follow-up period. For visualization purposes, a ROC curve was drawn at the landmark time points corresponding to the median follow-up for PFS and OS (i.e. 8.5 and 13.7, respectively) in months. Sensitivity, specificity, positive predictive value (PPV) and negative predictive value (NPV) were computed with the *SeSpPPVNPV* function available in the 'timeROC' R package, imposing the signature cutpoint of 0.007481 as defined in the training set.

Decision curve analysis (DCA): This was used to examine the net benefit of the miRNA model for predicting PFS. The method establishes the ability of a prognostic model to be clinically useful. The net benefit was calculated using the 'dca' package in R, available at https://www.mskcc.org/departments/epidemiology-biostatistics/biostatistics/decision-curve-analysis (Vickers 2006). Specifically, in this framework, the net benefit = true positive rate – (false positive rate x weighting factor); for which the weighting factor = threshold probability/(1 – threshold probability). This DCA was applied at landmark time points corresponding to the median follow-up duration (i.e., 8.5 months for PFS, and 13.7 months for OS).

Code Availability

No unreported custom computer code or algorithm was used to generate the results of this manuscript. The references of all source codes are included within Supplementary Methods section.

References

Abu Zohair, L.M. Prediction of Student's performance by modelling small dataset size. Int J Educ Technol High Educ 16, 27 (2019). https://doi.org/10.1186/s41239-019-0160-3

Appierto V, Callari M, Cavadini E, et al. A lipemia-independent NanoDrop(®)-based score to identify hemolysis in plasma and serum samples. Bioanalysis. 2014;6(9):1215-1226. doi:10.4155/bio.13.344

Bair E, Tibshirani R. Semi-supervised methods to predict patient survival from gene expression data. PLoS Biol. 2004;2(4):E108. doi:10.1371/journal.pbio.0020108

Blanche P, Dartigues JF, Jacqmin-Gadda H. Estimating and comparing time-dependent areas under receiver operating characteristic curves for censored event times with competing risks. Stat Med. 2013 Dec 30;32(30):5381-97. doi: 10.1002/sim.5958. Epub 2013 Sep 12. PMID: 24027076.

Brier, G. W., 1950: Verification of forecasts expressed in terms of probability. *Mon. Wea. Rev.*, **78**, 1–3, https://doi.org/10.1175/1520-0493(1950)078<0001:VOFEIT>2.0.CO;2.

Kirschner MB, Edelman JJ, Kao SC, et al. The impact of hemolysis on cell-free microRNA biomarkers. Front Genet. 2013;4:94. Published 2013 May 24. doi:10.3389/fgene.2013.00094

Kohn MA, Senyak J. Sample Size Calculators [website]. UCSF CTSI. 20 December 2021. Available at https://www.sample-size.net/ [Accessed 07 January 2022]

Gerds TA, Schumacher M. Consistent estimation of the expected Brier score in general survival models with right-censored event times. Biom J. 2006 Dec;48(6):1029-40. doi: 10.1002/bimj.200610301. PMID: 17240660.

Gerds TA, Schumacher M. Efron-type measures of prediction error for survival analysis. Biometrics. 2007 Dec;63(4):1283-7. doi: 10.1111/j.1541-0420.2007.00832.x. Epub 2007 Jul 25. PMID: 17651459.

Heagerty PJ, Lumley T, Pepe MS. Time-dependent ROC curves for censored survival data and a diagnostic marker. Biometrics. 2000 Jun;56(2):337-44. doi: 10.1111/j.0006-341x.2000.00337.x. PMID: 10877287.

Irizarry RA, Hobbs B, Collin F, et al. Exploration, normalization, and summaries of high-density oligonucleotide array probe level data. Biostatistics. 2003 Apr;4(2):249-64. doi: 10.1093/biostatistics/4.2.249. PMID: 12925520.

Johnson WE, Li C, Rabinovic A. Adjusting batch effects in microarray expression data using empirical Bayes methods. Biostatistics. 2007 Jan;8(1):118-27. doi: 10.1093/biostatistics/kxj037. Epub 2006 Apr 21. PMID: 16632515.

López-Romero P. Pre-processing and differential expression analysis of Agilent microRNA arrays using the AgiMicroRna Bioconductor library. BMC Genomics. 2011;12:64. Published 2011 Jan 26. doi:10.1186/1471-2164-12-64

Martínez-Camblor P, Pardo-Fernández JC. Smooth time-dependent receiver operating characteristic curve estimators. Stat Methods Med Res. 2018 Mar;27(3):651-674. doi: 10.1177/0962280217740786. Epub 2017 Nov 29. PMID: 29187044.

Massimino M, Biassoni V, Miceli R, Schiavello E, Warmuth-Metz M, Modena P, Casanova M, Pecori E, Giangaspero F, Antonelli M, Buttarelli FR, Potepan P, Pollo B, Nunziata R, Spreafico F, Podda M, Anichini A, Clerici CA, Sardi I, De Cecco L, Bode U, Bach F, Gandola L. Results of nimotuzumab and vinorelbine, radiation and re-irradiation for diffuse pontine glioma in childhood. J Neurooncol. 2014 Jun;118(2):305-312. doi: 10.1007/s11060-014-1428-z. Epub 2014 Apr 3

Mogensen UB, Ishwaran H, Gerds TA. Evaluating random forests for survival analysis using prediction error curves. J Stat Softw. 2012 Sep;50(11):1-23. doi: 10.18637/jss.v050.i11. PMID: 25317082; PMCID: PMC4194196.

Oller, R., and K. Langohr. 2017. FHtest: an R package for the comparison of survival curves with censored data. J. Stat. Softw. 81(15):1–25. DOI:10.18637/JSS.V081.I15

Porzelius C, Binder H, Schumacher M. Parallelized prediction error estimation for evaluation of high-dimensional models. Bioinformatics. 2009 Mar 15;25(6):827-9. doi: 10.1093/bioinformatics/btp062. Epub 2009 Jan 28. PMID: 19176556.

Simon R., Lam A., Li M., et al. Analysis of gene expression data using BRB-ArrayTools. Cancer Informatics, 3 (2007), pp. 11-17 https://doi.org/10.1177/117693510700300022

Therneau T (2020). A Package for Survival Analysis in R. R package version 3.2-3, https://CRAN.R-project.org/package=survival.

Vickers AJ, Elkin EB. Decision curve analysis: a novel method for evaluating prediction models. Med Decis Making. 2006;26(6):565-574. doi:10.1177/0272989X06295361

Wessa, P. (2020), Free Statistics Software, Office for Research Development and Education, version 1.2.1, URL https://www.wessa.net/
