# Supplementary material for: A microRNA Prognostic Signature in Patients with Diffuse Intrinsic Pontine Gliomas through Non-Invasive Liquid Biopsy
Source: Cancers (Basel). 2022 Sep 2;14(17):4307. doi: 10.3390/cancers14174307 (PMC9454461; doi:10.3390/cancers14174307)
Supplement: Supplementary file 1 [file cancers-14-04307-s001.zip › edited-Supplementary Results_rev.pdf]

Supplementary Results

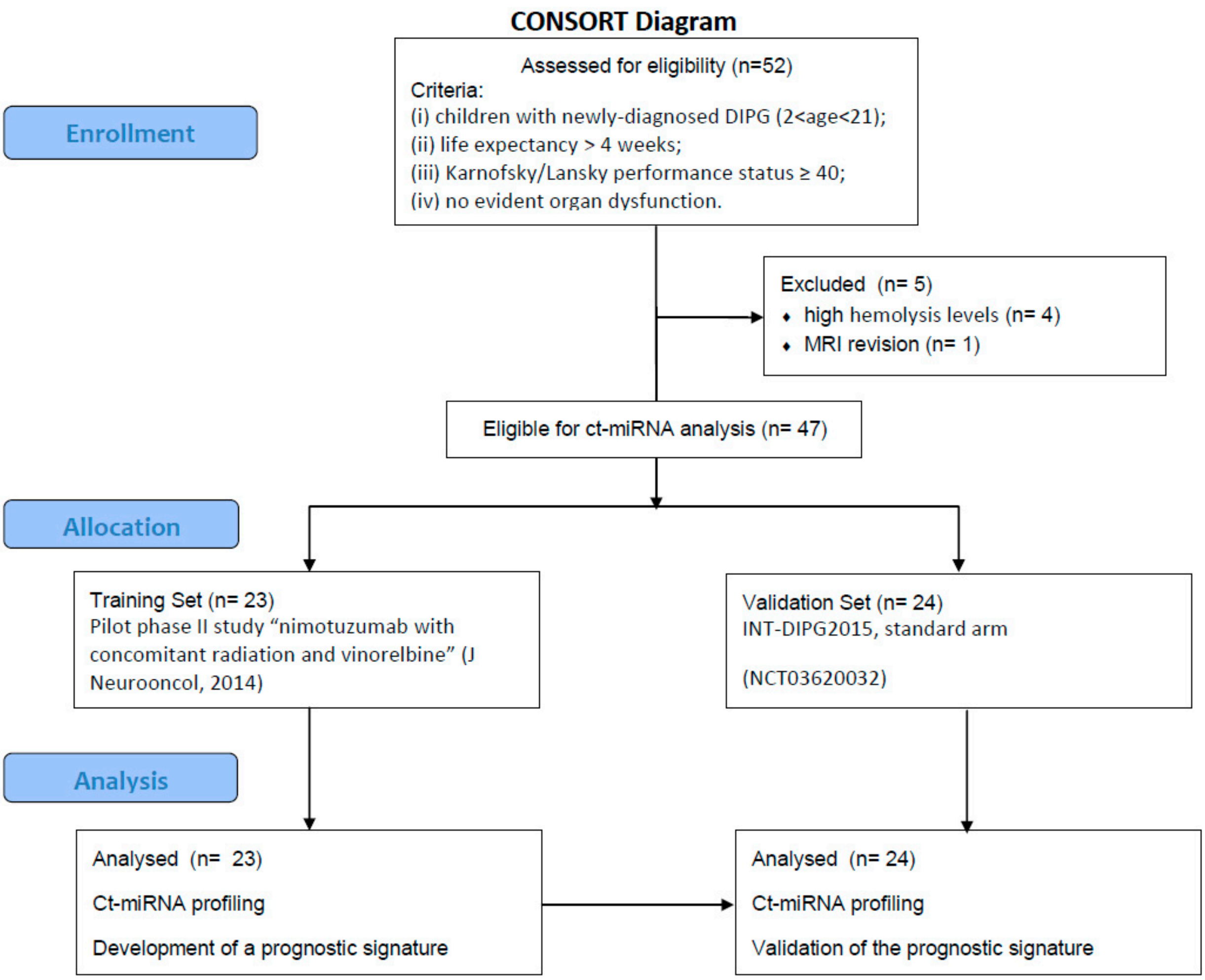

Figure S1: Consort Flow Diagram

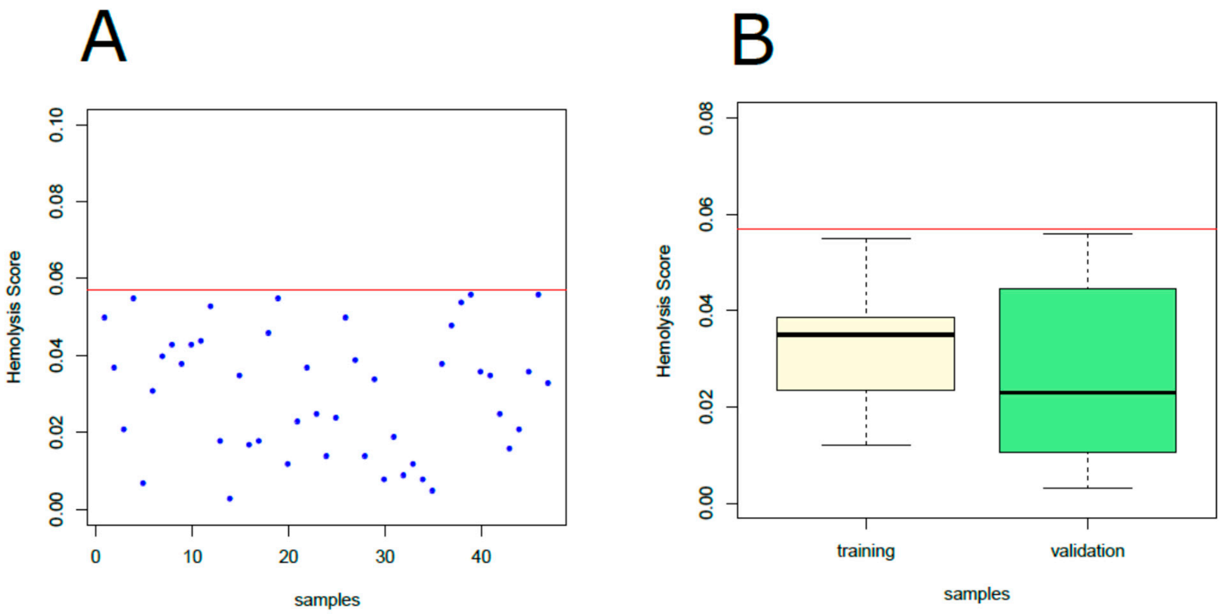

Figure S2. Hemolysis scores **(**A) Hemolysis scores of DIPG patients. Serum samples used in this study were checked for the absence of hemolysis. Ultraviolet-visible absorbance (A414 and A385) was measured by NanoDrop One spectrophotometer. The plot shows the resulting hemolysis scores (Appierto et al., 2014) for the total n = 47 samples comprising the training and validation data sets pooled. The red line denotes the threshold (hemolysis score = 0.057), following Appierto et al. (2014), indicating low levels of hemolysis. (B) Hemolysis in the training and validation data sets. Boxplots of their hemolysis scores suggest no significant differences (P = 0.268 Mann-Whitney U-Test; median of the training set = 0.035, median of the validation set = 0.023).

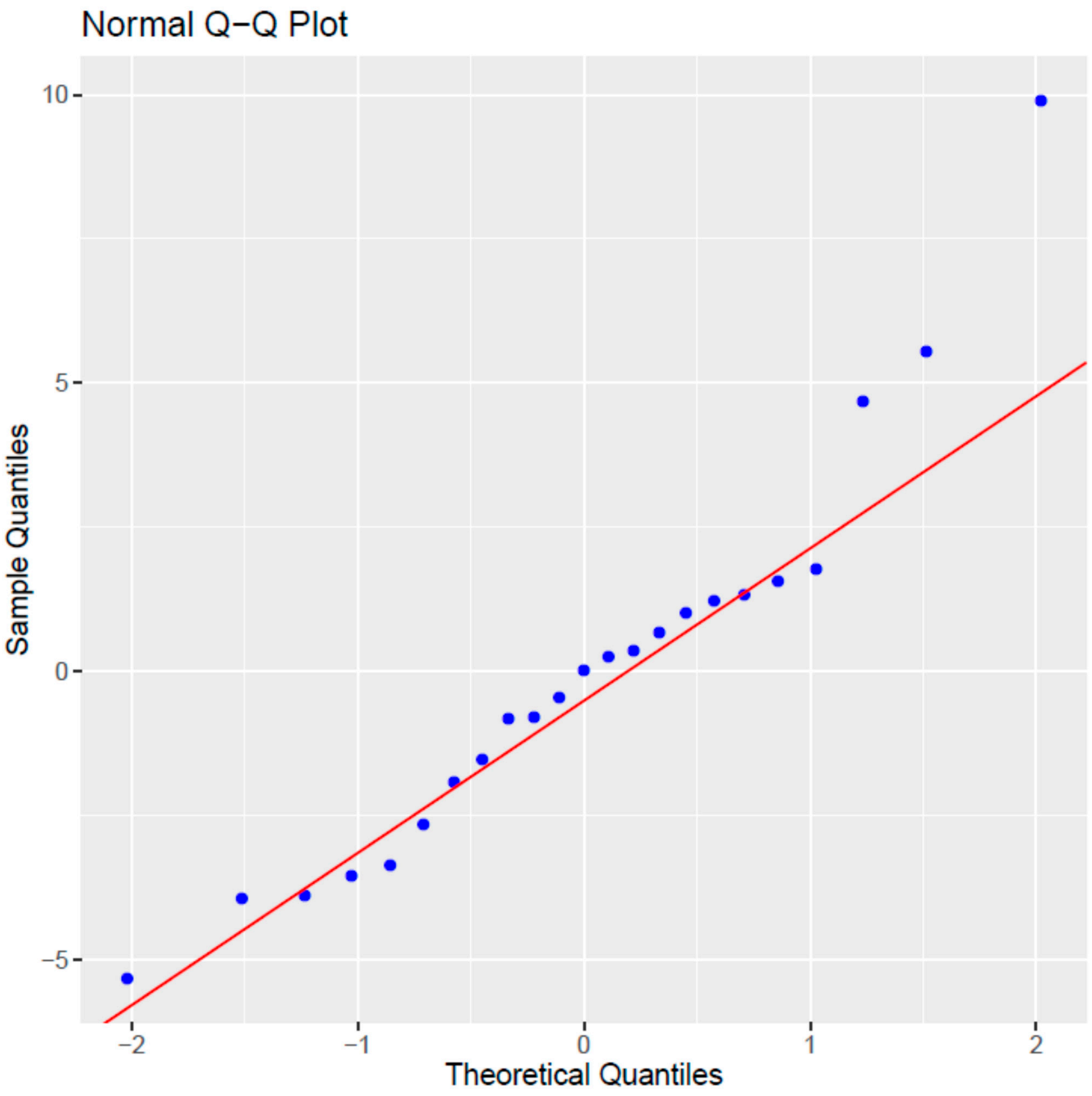

Figure S3. Graph for detecting violation of normality assumption. The Normal Q-Q plot was drawn using the *ols_plot_resid_qq* function in 'olsrr' package. This plot examines whether the residuals are normally distributed: the theoretical percentiles of the normal distribution on the x axis and the sample percentiles of the residuals on the y axis. The relationship between the theoretical percentiles and the sample percentiles is approximately linear. Therefore, the normal probability plot of the residuals suggests that the error terms are indeed normally distributed

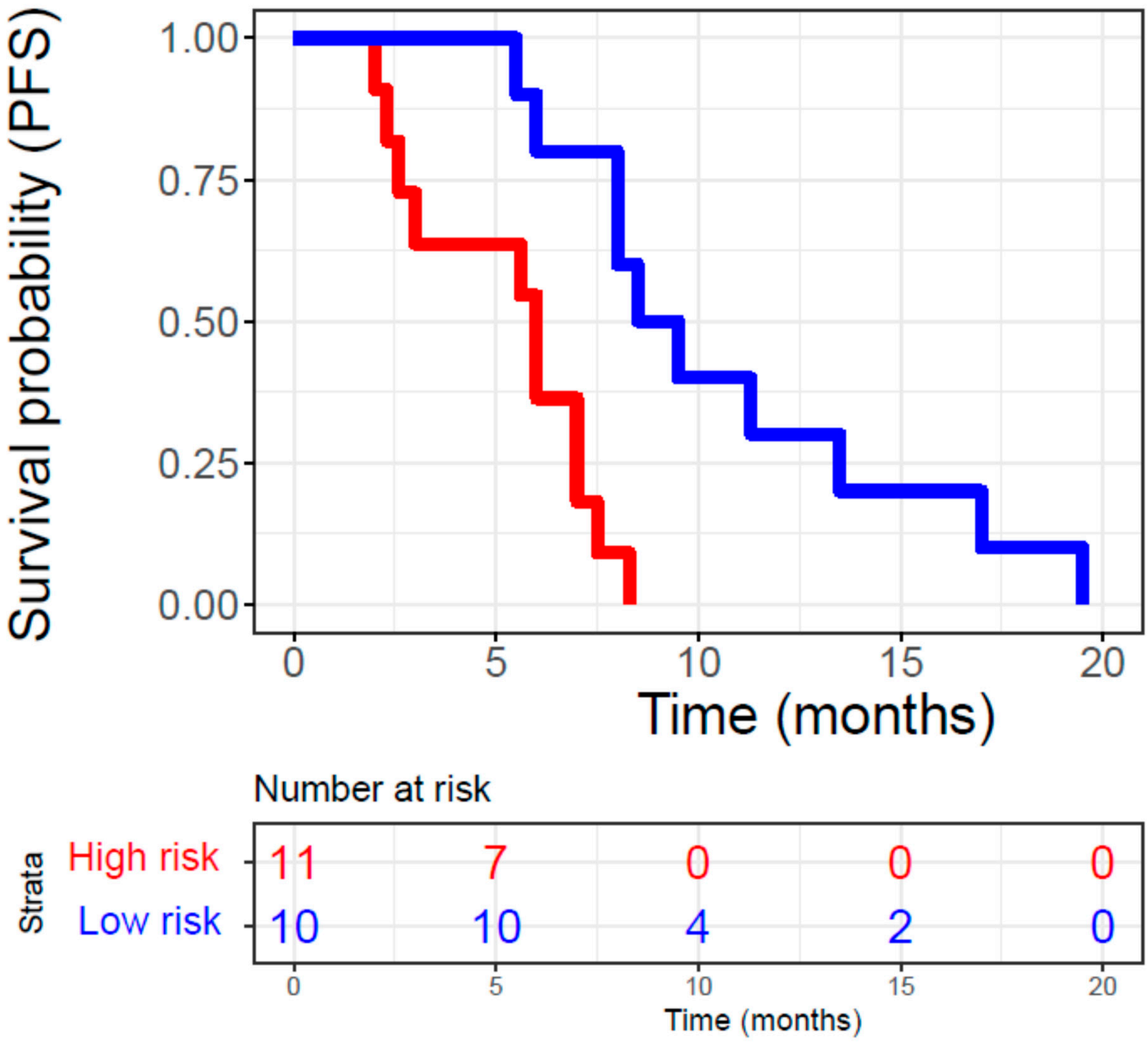

Figure S4 Kaplan–Meier survival curves excluding the long-term survivors with DIPG. The value of our risk stratification according to the ct-miRNA model was tested excluding two patients (long-term survivors). PFS was the clinical endpoint. High-risk patients (n = 11) had a shorter survival than did low-risk patients (n = 10) [log-rank, P = 0.00079, hazard ratio (HR) = 6.42, 95% confidence interval (CI) 1.82–21.51; Fleming–Harrington test, P = 0.0056], whose median PFS was 6 and 9 months, respectively. High-risk in red, low-risk in blue.

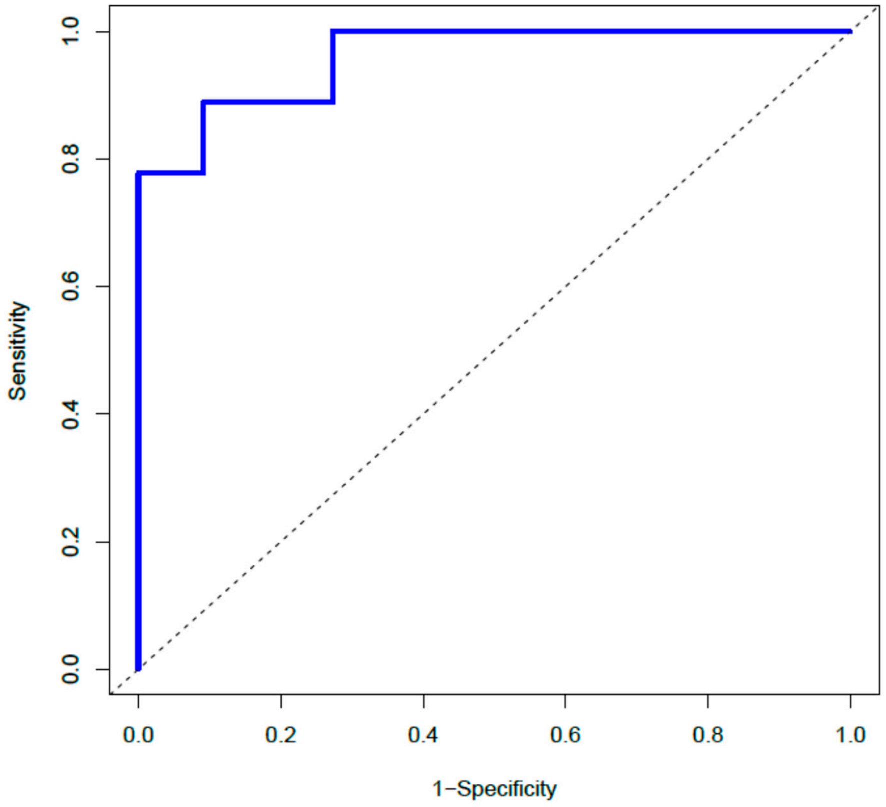

Figure S5. Receiver-operator characteristic (ROC) curve for DIPG patients' outcome, at 8.5 months follow-up, for the fitted ct-miRNA model (AUC = 0.96; 95% CI: 0.888–1).

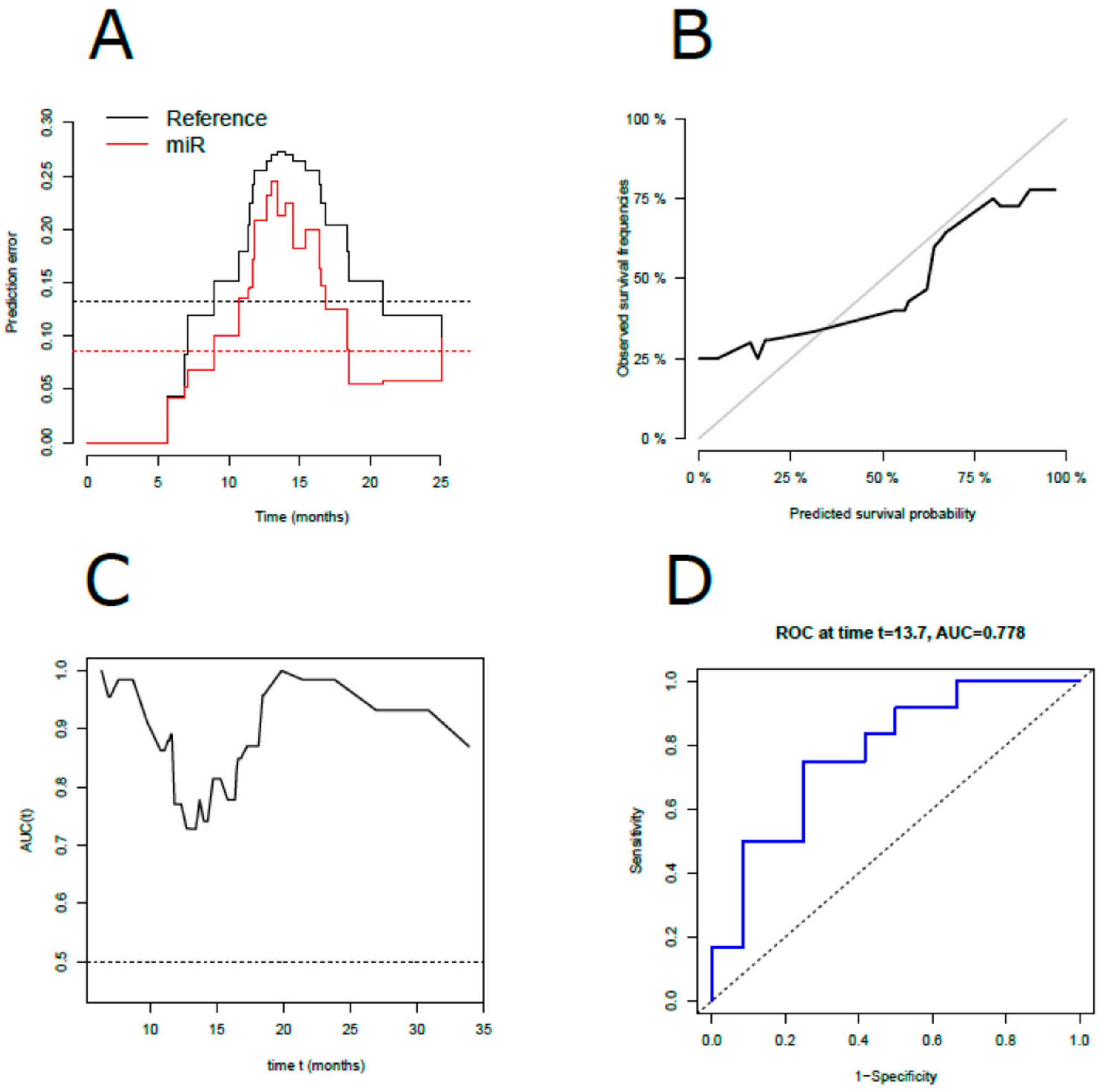

Figure S6

Performance assessment based on OS

(A) Prediction error curves using Brier scores based on the ct-miRNA signature's stratification as well as on estimates for all patients without any stratification (reference curve). The IBS over the interval from 0 to 25 months was 0.086 for the ct-miRNA signature, and 0.132 for the reference. At the landmark time point of 13.7 months, the leave-one-out cross-validation estimate was 0.213 for the ct-miRNA model, and 0.272 for the reference. Red dotted line= IBS for the ct-miRNA model; black dotted line= IBS for the reference model.

(B) Calibration plot for OS at the landmark time of 13.7 months. The plot shows the predictions obtained by the model on the x-axis and the observed outcome on the y-axis.

(C) Area under the ROC curves (AUC) based on our ct-miRNA model fitting the OS obtained by a time-dependent ROC analysis. Time (months).

(D) Receiver-operator characteristic (ROC) curve for outcome at 13.7 months for the ct-miRNA model (AUC=0.78; 95% CI: 0.591-0.965). The sensitivity, specificity, PPV, and NPV were 71.4%, 80%, 75%, 75%, respectively.
